# Supplementary material for: Modeling glioblastoma heterogeneity as a dynamic network of cell states
Source: Mol Syst Biol. 2021 Sep 16;17(9):e10105. doi: 10.15252/msb.202010105 (PMC8444284; doi:10.15252/msb.202010105)
Supplement: Supplementary file 6 — Source Data for Figure 5 [file MSB-17-e10105-s004.zip › Figure5A_sourcedata/GSEA_3071/hallmarks_stateII.GseaPreranked.1623742536692/HALLMARK_P53_PATHWAY.html]

Details for gene set HALLMARK\_P53\_PATHWAY[GSEA]

|  || Dataset | state23071 |
| Phenotype | NoPhenotypeAvailable |
| Upregulated in class | na\_neg |
| GeneSet | HALLMARK\_P53\_PATHWAY |
| Enrichment Score (ES) | -0.22523683 |
| Normalized Enrichment Score (NES) | -1.1371429 |
| Nominal p-value | 0.2757417 |
| FDR q-value | 0.5998751 |
| FWER p-Value | 1.0 |
Table: GSEA Results Summary

  

Fig 1: Enrichment plot: HALLMARK\_P53\_PATHWAY      
 Profile of the Running ES Score & Positions of GeneSet Members on the Rank Ordered List

  

| PROBE | GENE SYMBOL | GENE\_TITLE | RANK IN GENE LIST | RANK METRIC SCORE | RUNNING ES | CORE ENRICHMENT || 1 | KRT17 |  |  | 47 | 1.256 | 0.0210 | No |
| 2 | PCNA |  |  | 58 | 1.214 | 0.0500 | No |
| 3 | LIF |  |  | 64 | 1.184 | 0.0796 | No |
| 4 | JAG2 |  |  | 313 | 0.743 | 0.0373 | No |
| 5 | CCND3 |  |  | 390 | 0.683 | 0.0362 | No |
| 6 | NUDT15 |  |  | 461 | 0.646 | 0.0356 | No |
| 7 | POLH |  |  | 487 | 0.635 | 0.0459 | No |
| 8 | PERP |  |  | 496 | 0.630 | 0.0603 | No |
| 9 | TM4SF1 |  |  | 512 | 0.624 | 0.0728 | No |
| 10 | SPHK1 |  |  | 551 | 0.603 | 0.0790 | No |
| 11 | SOCS1 |  |  | 593 | 0.581 | 0.0840 | No |
| 12 | IER5 |  |  | 686 | 0.536 | 0.0751 | No |
| 13 | EPHA2 |  |  | 791 | 0.502 | 0.0623 | No |
| 14 | GADD45A |  |  | 867 | 0.480 | 0.0561 | No |
| 15 | SDC1 |  |  | 872 | 0.479 | 0.0676 | No |
| 16 | PTPN14 |  |  | 964 | 0.454 | 0.0568 | No |
| 17 | RAD51C |  |  | 967 | 0.453 | 0.0681 | No |
| 18 | HBEGF |  |  | 972 | 0.451 | 0.0788 | No |
| 19 | SLC7A11 |  |  | 998 | 0.445 | 0.0842 | No |
| 20 | IER3 |  |  | 1010 | 0.442 | 0.0929 | No |
| 21 | CDKN1A |  |  | 1088 | 0.419 | 0.0847 | No |
| 22 | TPRKB |  |  | 1168 | 0.403 | 0.0756 | No |
| 23 | DDB2 |  |  | 1189 | 0.399 | 0.0810 | No |
| 24 | SLC3A2 |  |  | 1203 | 0.396 | 0.0880 | No |
| 25 | PPP1R15A |  |  | 1284 | 0.381 | 0.0781 | No |
| 26 | DNTTIP2 |  |  | 1478 | 0.343 | 0.0391 | No |
| 27 | HRAS |  |  | 1538 | 0.334 | 0.0331 | No |
| 28 | STOM |  |  | 1594 | 0.325 | 0.0279 | No |
| 29 | JUN |  |  | 1725 | 0.307 | 0.0036 | No |
| 30 | PROCR |  |  | 1757 | 0.304 | 0.0038 | No |
| 31 | S100A10 |  |  | 1852 | 0.292 | -0.0119 | No |
| 32 | AEN |  |  | 1913 | 0.284 | -0.0195 | No |
| 33 | TXNIP |  |  | 1918 | 0.283 | -0.0131 | No |
| 34 | IRAK1 |  |  | 1943 | 0.280 | -0.0118 | No |
| 35 | PMM1 |  |  | 1976 | 0.276 | -0.0126 | No |
| 36 | CASP1 |  |  | 1994 | 0.273 | -0.0097 | No |
| 37 | DRAM1 |  |  | 2174 | 0.255 | -0.0475 | No |
| 38 | HSPA4L |  |  | 2203 | 0.252 | -0.0479 | No |
| 39 | RHBDF2 |  |  | 2214 | 0.250 | -0.0439 | No |
| 40 | PTPRE |  |  | 2265 | -0.255 | -0.0497 | No |
| 41 | KIF13B |  |  | 2294 | -0.258 | -0.0499 | No |
| 42 | HEXIM1 |  |  | 2428 | -0.274 | -0.0758 | No |
| 43 | AK1 |  |  | 2446 | -0.276 | -0.0728 | No |
| 44 | XPC |  |  | 2518 | -0.288 | -0.0830 | No |
| 45 | TOB1 |  |  | 2580 | -0.298 | -0.0904 | No |
| 46 | RALGDS |  |  | 2775 | -0.331 | -0.1299 | No |
| 47 | CDKN2B |  |  | 3046 | -0.384 | -0.1869 | No |
| 48 | ABAT |  |  | 3075 | -0.391 | -0.1837 | No |
| 49 | ISCU |  |  | 3101 | -0.399 | -0.1796 | No |
| 50 | CD81 |  |  | 3196 | -0.427 | -0.1918 | No |
| 51 | NOTCH1 |  |  | 3202 | -0.429 | -0.1819 | No |
| 52 | CTSD |  |  | 3227 | -0.435 | -0.1765 | No |
| 53 | CYFIP2 |  |  | 3265 | -0.445 | -0.1742 | No |
| 54 | ZMAT3 |  |  | 3380 | -0.490 | -0.1897 | No |
| 55 | PRMT2 |  |  | 3483 | -0.534 | -0.2012 | No |
| 56 | WWP1 |  |  | 3581 | -0.592 | -0.2098 | Yes |
| 57 | FOXO3 |  |  | 3594 | -0.601 | -0.1972 | Yes |
| 58 | FBXW7 |  |  | 3629 | -0.626 | -0.1894 | Yes |
| 59 | SAT1 |  |  | 3659 | -0.653 | -0.1796 | Yes |
| 60 | IP6K2 |  |  | 3689 | -0.672 | -0.1694 | Yes |
| 61 | NUPR1 |  |  | 3825 | -0.813 | -0.1817 | Yes |
| 62 | TSC22D1 |  |  | 3872 | -0.887 | -0.1701 | Yes |
| 63 | CDH13 |  |  | 3908 | -0.963 | -0.1538 | Yes |
| 64 | FOS |  |  | 3911 | -0.966 | -0.1292 | Yes |
| 65 | BTG2 |  |  | 3917 | -0.999 | -0.1044 | Yes |
| 66 | NINJ1 |  |  | 3929 | -1.015 | -0.0808 | Yes |
| 67 | CD82 |  |  | 3973 | -1.119 | -0.0624 | Yes |
| 68 | APP |  |  | 3982 | -1.164 | -0.0341 | Yes |
| 69 | MXD4 |  |  | 3985 | -1.174 | -0.0041 | Yes |
| 70 | RGS16 |  |  | 3996 | -1.227 | 0.0253 | Yes |
Table: GSEA details [plain text format]

  

Fig 2: HALLMARK\_P53\_PATHWAY: Random ES distribution      
 Gene set null distribution of ES for **HALLMARK\_P53\_PATHWAY**
